# The ARF GTPase regulatory network in collective invasion and metastasis

**DOI:** 10.1042/BST20221355

**Published:** 2023-08-25

**Authors:** Konstantina Nikolatou, David M. Bryant, Emma Sandilands

**Affiliations:** 1 School of Cancer Sciences, University of Glasgow, Glasgow G61 1HQ, U.K.; 2 The CRUK Beatson Institute, Glasgow G61 1BD, U.K.

**Keywords:** ARF GTPases, cell polarity, invasion, metastasis, network analysis

## Abstract

The ability to remodel and move cellular membranes, and the cargoes regulated by these membranes, allows for specialised functions to occur in distinct regions of the cell in a process known as cellular polarisation. The ability to collectively co-ordinate such polarisation between cells allows for the genesis of multicellularity, such as the formation of organs. During tumourigenesis, the rules for such tissue polarisation become dysregulated, allowing for collective polarity rearrangements that can drive metastasis. In this review, we focus on how membrane trafficking underpins collective cell invasion and metastasis in cancer. We examine this through the lens of the ADP-ribosylation factor (ARF) subfamily of small GTPases, focusing on how the ARF regulatory network — ARF activators, inactivators, effectors, and modifications — controls ARF GTPase function.

## Introduction

**Figure 1.**
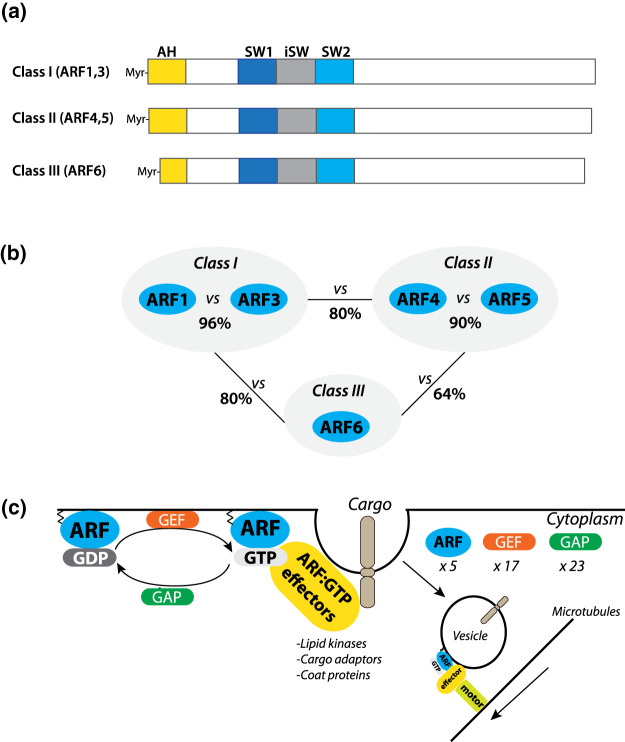
ARF GTPase domain structure, homology, and function. (**a**) Domain structure and (**b**) homology between Class I (ARF1, 3), Class II (ARF4, ARF5), and Class III (ARF6) ARF GTPases in humans. AH, amphipathic helix; SW1, switch 1; iSW, interswitch; SW2, switch 2. Myr, myristoylated. (**c**) ARF GTPases are GTP-loaded by GTP Exchange Factors (GEFs), while nucleotide is hydrolysed to GDP by GTPase-activating proteins (GAPs). At the membrane, regulated by the myristoylation of the N-terminus, ARF GTPases bind many effector proteins, including lipid kinases, cargo adaptors, and coat proteins. On the endosome, ARF can also regulate interaction with molecular motors to transport vesicles along the cytoskeleton. In human, there are 5 ARF GTPases (in blue), 17 GEFs (orange), and 23 GAPs (in green). ARF-GTP effectors, yellow.

The ADP-ribosylation factors (ARFs) are a group of small GTPases, of which there are five in humans [1]. All ARF GTPases consist of a minimally divergent sequence and structure, comprised of an N-terminal amphipathic helix, guanine nucleotide-binding switch region (Switch 1, Interswitch, and Switch 2 regions), and variable C-terminal regions (Figure 1a). ARF GTPases are divided into three groups, based on sequence homology: Class I (ARF1, ARF3; the ARF2 GTPase was lost in humans), Class II (ARF4, ARF5), and the sole Class III member ARF6 (Figure 1a,b). Like all Ras superfamily small GTPases, ARF association with effector proteins is controlled by their GTPase cycle [2,3]. GTP loading of ARFs is controlled by GTPase exchange factors (GEFs), while the inherent GTP hydrolysis activity of ARFs is potentiated by GTPase-activating proteins (GAPs), which hydrolyse GTP into GDP (Figure 1c). All ARF GTPases are N-myristoylated, which allows for association with membranes. Upon GTP binding, the ARF N-terminal amphipathic helix is inserted in the membrane and interactions with membrane phospholipids are stabilised, ensuring ARF activation occurs close to the membrane surface.

ARF GTPases function through the recruitment of effector proteins, such as lipid kinases and phosphatases to modify the phospholipid content of a domain, cargo adaptor proteins to sort proteins into budding vesicles, vesicle coat proteins to allow a membrane carrier to bud from a membrane into a vesicle, and molecular motors to allow vesicle transport to new destinations [2,3] (Figure 1c). Given that the asymmetric localisation of proteins and membrane identity are essential events that underpin cellular polarisation, by extrapolation, this suggests that ARF GTPases may be key players in cellular polarity by controlling where cargo proteins are loaded into domains for sorting into other cellular locations.

In this review, we focus on what we still do not know about the ARF GTPases, particularly relating to the ARF regulatory network — their activators, inactivators, effectors, and modifications. This is aimed at highlighting how consideration of the network surrounding ARF GTPases is essential in unravelling their functions. Moreover, we focus on how systematic knockdown or knockout of GEFs, GAPs, and effectors with ARF GTPases can identify co-acting partners, which may not easily be inferred from biochemical data alone. The cell and structural biology of ARF GTPase function in membrane trafficking has been extensively reviewed elsewhere, and we point readers to this excellent literature for an in-depth summary of what is known about ARF GTPases [1,4–9]. Moreover, although many key studies have indicated that ARF GTPases can be inhibited *in vivo* to perturb tumour growth and metastasis, we do not review these studies here, but instead direct readers to additional review materials [10–13].

## What are the localisations and sites of function of ARF GTPases?

In the classical view of ARF GTPases, Class I and II ARFs act in biosynthetic trafficking, localising primarily to the Golgi apparatus and endosomal structures [2,3]. In contrast, the sole Class III ARF, ARF6, localises primarily to the plasma membrane and endocytic compartments, and has been ascribed a function in endocytosis and recycling. However, all ARFs occur at membrane locations outside of these locales, suggesting a wider repertoire of ARF GTPase function. For instance, in addition to clear roles for ARF1 at the Golgi apparatus [2,3], ARF1 can function in dynamin-independent endocytosis [14], controlling adaptor complex recruitment and sorting in early endocytic compartments [15], at recycling endosomes to regulate retrograde transport to the trans-Golgi network (TGN) [16,17], between the endoplasmic reticulum (ER) and TGN [18], and at mitochondria-ER contact sites [19,20]. Similarly, ARF4 functions at the ER-Golgi intermediate compartment (ERGIC) [21], at recycling endosomes [17], in endocytosis at the cell surface [22], and delivery of ciliary cargo [23,24]. These myriad studies reporting distinct localisations suggest that ARF GTPases function in wide-ranging membrane transport processes.

Due to the high sequence conservation within and between Class I and Class II ARF GTPases (Figure 1b), one open question is the extent to which these proteins have distinct or redundant functions. Loss of function (knockdown and knockout) studies examining cargo trafficking in single cells suggests that ARFs have shared, co-operative functions [25,26]. To this end, recent elegant work from the Botanelli Laboratory, using endogenous tagging of Class I and II ARFs, demonstrates that even within an organelle or membrane carrier, while functioning in a co-ordinated fashion, each ARF defines segregated nanodomains [21]. This raises the tantalising possibility that ARFs do indeed co-operate but may regulate distinct stages of cargo sorting and/or transport. What remains to be demonstrated is how each ARF is differentially recruited to such distinct domains on the same organelle or carrier.

## What controls ARF GTPase expression and are there modifications that control function?

Very little is known about what controls the expression of ARF GTPases. One possible explanation for this could be a general assumption that ARF GTPases are ubiquitous regulators of membrane traffic. The Human Protein Atlas [27] reveals, however, that while all ARFs are ubiquitously expressed, variation in expression levels between organs exists, such as elevation of Class I ARF expression in the brain and neural tissues. Unlike Class II and III ARFs, both Class I ARF GTPases are encoded from multiple transcripts that almost exclusively result in identical protein but differ in transcript 5′- and 3′-untranslated regions (UTRs) (ARF1, 4 transcripts; ARF3, 14 transcripts; [28]). This suggests that there may be key regulatory mechanisms controlling Class I ARF expression that we do not yet know. Moreover, the factors that induce transcription of all ARFs are mostly unknown, except for the case of CREB3 transcription factor-driven up-regulation of ARF4 in instances of Golgi stress [29].

We also know very little about the modifications that may regulate ARF GTPase function. Ubiquitination was reported to control the turnover of GTP-loaded ARF6 [30], yet the mutations used to map this site also perturb membrane association [31], keeping the question open as to how ubiquitination controls ARF6 function. This is a largely unexplored area in ARF GTPases, even though publicly available mass spectrometry studies identify ubiquitination of all ARF GTPases, including ubiquitination of residues in the nucleotide and effector-binding regions [32]. How exactly ARF ubiquitination controls cellular function remains to be explored. Moreover, these same databases identify acetylation events outside the canonical N-terminal acetylation, as well as phosphorylation on tyrosine, serine, and threonine residues [32], but the consequences of these are unknown. Collectively, this indicates that we have only begun to scratch the surface of how modifications of ARF GTPases contribute to their function.

## How are ARF GEFs, GAPs, and effectors controlled, and how do they regulate ARFs?

The highly conserved nature of the ARF GEF (Sec7) and ARF GAP domains, from yeast to humans, allowed the identification of 17 GEFs and 23 GAPs in human [1]. The structures, domains and proposed specificities of these regulators have been excellently reviewed elsewhere [1,4–9]. What we don't know is the extent to which the exchange or hydrolysis of nucleotides in ARFs can be catalysed by non-canonical GEFs or GAPs. For instance, AMP-activated protein kinase (AMPK) can function as a non-canonical, non-Sec7 domain-containing GEF for Arf6 [33,34]. These GTPases can, for instance, even act as GEFs for other GTPases, such as ARL13, in cooperation with BART, acting as a GEF for ARL3 [35–37]. As a further example, ELMO domain (ELMOD) proteins are non-canonical GAPs working both on ARF and ARL GTPases [38,39]. Although preferential catalytic activity of these factors has been proposed for different ARFs, identifying how even canonical GEFs and GAPs work with ARF GTPases, or the extent to which these work additionally or preferentially on ARLs is unclear.

### GEF or GAP specificity for distinct ARF GTPases

One key consideration is that part of the activation mechanism (GTP loading) of ARF GTPases involves close association with membranes. Historically, many ARF GEF and GAP preferences for ARF GTPases have been elucidated from *in vitro* biochemical assays. Here, ARFs with the amphipathic helix deleted have often been used, which allows nucleotide exchange reactions to occur in solution without membrane, stimulated with the minimal exchange-inducing region (such as the Sec7-PH domains) of a GEF. These assays often reveal that exchange activity can occur from most ARF GEFs on most ARFs, but a preference for a particular class of ARF is observed. An example of this is represented in the naming of the PSD/Exchange Factor for ARF6 (EFA6) subgroup of GEFs for their *in vitro* preference for ARF6 [40]. However, the amphipathic helix of ARFs comprises a considerable fraction of the divergent sequence between ARFs, questioning if GEF-ARF specificities may change when membranes are considered or in cells. Indeed PSD/EFA6 shows robust activation of the Class I ARF, ARF1, when membranes are included in *in vitro* assays [41], and a preference for the Class I ARF GTPase ARF3, and not ARF6, in invasive prostate cancer cell lysates [42]. Collectively, this poses a problem for extrapolating potentially co-acting GEF-GAP-ARF-Effector modules from classical biochemical data. Indeed, two recent proteomic studies examining the repertoire of what GTP-loaded ARFs can bind to indicate a highly, but not completely, overlapping effector set [43,44]. If, stated crudely, most things can bind most things in this ARF-centred network, how is specificity in ARF GTPase signalling generated? Moreover, most GEFs and GAP are multidomain proteins that link and integrate ARF activation with other signalling pathways, or aid in the recruitment of effectors and co-acting modules [1]. Without diminishing the power and necessity of *in vitro* validation of activity, such co-operativity is overlooked when using isolated domains in a membrane-free environment.

### Phenotypic screening to identify co-acting ARF regulatory modules

**Figure 2.**
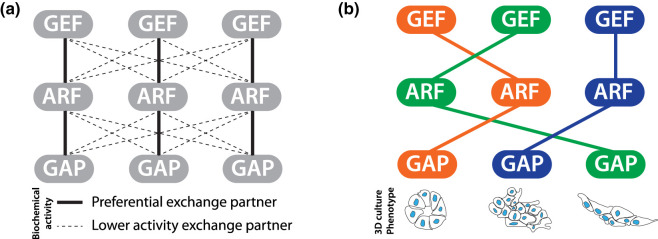
Mapping co-acting ARF GTPase modules. (**a**) Identifying potentially co-acting ARF GEFs and GAPs based on biochemical activity is complicated by having some level of enzymatic activity on most ARF GTPases. Lines, interactions based on activities in solution *in vitro*. Thick line, preferential pairing. Thin dotted lines, lower activity exchange partners. (**b**) Phenotypic screening offers an advantage of determining which GEFs and GAPs show phenotypes similar to each ARF GTPase.

Given the theoretic potential for almost any combination of ARF, GEF, GAP, and effector to co-operate (Figure 2a), we performed an unbiased phenotypic screen to identify which components in the ARF regulatory network, upon loss, resulted in a similar phenotype [42]. We reasoned that this would identifying co-operating modules. To do this, we developed a novel 3-dimensional (3D) culture-based screening of prostate cancer cells, live-imaged these cells over multiple days, and applied a machine-learning-based classification to quantify alternate resulting phenotypes. These alternate phenotypes included spheroids that stayed spherical, those that invaded into the extracellular matrix (ECM) only very locally, and those that formed highly invasive chains. Through arrayed shRNA screening (one manipulation per well, multiple validated shRNAs per gene), we could identify phenotypically similar gene knockdowns, and test whether these were functionally co-operating modules (Figure 2b).

We identified pairing of the GEF PSD/EFA6 as an exchange factor for the less-studied Class I ARF, ARF3 (and not ARF6 as the GEF namesake suggests) [42]. Moreover, we identified RAB11FIP4/Arfophilin-2, a dual Rab11- and ARF-binding protein that regulates endocytic recycling dynamics as an ARF3 effector [45,46]. Through cell biological approaches we could map the key cargo for ARF3 as the cell–cell adhesion protein N-cadherin. This PSD-ARF3-RAB11FIP4 module controlled the post-endocytic recycling of N-cadherin. Functionally, this resulted in a switch in the collective behaviours of prostate cancer cells. In cells with high levels of ARF3, N-cadherin total protein and cell surface levels were increased, concomitant with collective migration of a cell sheet *in vitro*, and only local metastases in intraprostatic xenograft of cells. Moreover, high levels of N-cadherin were associated with a decreased level of lymph-node metastasis in prostate cancer patients. In contrast, low levels of ARF3/N-cadherin were associated with invasion led by chain-type invasive modality, distal metastasis in xenograft models, and increased lymph-node metastasis in prostate cancer patients. Indeed, levels of N-cadherin could identify patients with best versus poorest outcome, dependent on whether ARF3 was expressed. This emphasises how using phenotypic screening allows for uncovering clinically relevant, unexpected ARF complexes.

The PSD-ARF3-RAB11FIP4 module is but one potential complex that could be identified from our approach of phenotypic screening [42]. This resource potentially has a wealth of additional modules yet to be identified. However, it must be stated that one limitation of any high-throughput approach is that a lack of co-clustering of actually co-operating modules could indeed be from a bona fide lack of phenotype, or from a lack of robust effect across replicates. Such limitations, and the limitation of the screen only being performed in a single cell type, wherein different modifications of the ARFome are likely to occur in different cells, means there is still much to uncover.

### Modifications of the ARF regulatory network

A parallel approach to map how ARF GTPases are regulated is through mass spectrometry approaches, such as through the fusion of ARFs to promiscuous biotin ligases (BioID, TurboID) to identify ARF-proximal proteins [43,44]. Approaches using GTP-preferential mutants of ARFs have allowed mapping of the repertoire of what ARFs can bind to. A challenge, as aforementioned, is instead determining what ARFs modules actually occur during a morphogenetic event.

**Figure 3.**
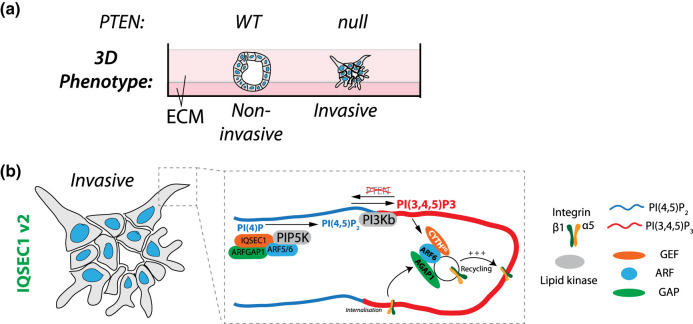
Cellular function of ARF GTPases in collective cell invasion using 3D culture. (**a**) Cartoon of alternate phenotypes that can arise when cancer cells are grown inside ECM gels, wherein PTEN presence (WT) versus absence (null) can influence whether cells are non-invasive or collectively invasive. (**b**) Model for select members of the ARF regulome in collective cell invasion. IQSEC1, in combination with ARF5/6 and the GAP ArfGAP1, regulate the PI3Kβ-dependent production of PI(3,4,5)P_3_ at the front of invasive protrusions, by promoting PI(4,5)P_2_ production through the ARF-GTP lipid kinase effector, PIP5K1B. Active α5β1-integrins drive invasion by being recycled to invasive protrusion tips in response to PI(3,4,5)P_3_. This occurs through ARF6-AGAP1 activity, which is promoted by the PI(3,4,5)P_3_-binding CYTH2^2G^ ARF GEF.

We recently used ARF6-TurboID (wild-type ARF6, not a GTP-preferential mutant) in high-grade serous ovarian cancer (HGSOC) cells to map the ARF6 interactome across an isogenic series of loss of TP53 and PTEN, alone or in combination [47]. In HGSOC, the loss of TP53 and PTEN results in aggressive tumourigenesis and some level of treatment resistance in both patients and murine models [48]. In *in vitro* 3D culture, this manifests as collective invasion from spheroids into ECM (Figure 3a). We showed that depletion of ARF6 reverses the effect of PTEN loss, suggesting that ARF6-dependent functions are essential for the invasive activity normally suppressed by PTEN. TurboID-based profiling of ARF6 interactors, coupled to a CRISPR-mediated proteomic screen to identify the key regulators of invasion, revealed the α5β1-integrin pair as the key cargo of this module, in line with previous reports of ARF5 and ARF6 requirement for α5β1-integrin trafficking [49]. Moreover, this identified a single GEF, Cytohesin-2/CYTH2/ARNO, and single GAP, AGAP1, that co-operate with ARF6 to regulate invasion. By identifying exactly the combination of GEF-ARF-GAP that regulates invasion, we could use combined CYTH2-ARF6-AGAP1 expression in HGSOC patients to identify those that have the poorest survival and clinical features.

Many ARF GEFs and GAPs can be extensively spliced, generating isoforms with distinct domains and therefore different interactors and regulatory inputs. Much of this additional complexity layer has largely been neglected but provides an opportunity to both identify and potentially therapeutically target pathogenic regulation of ARF GTPases, while sparing normal function. An important caution, therefore, is to ensure when generating or evaluating knockouts or knockdowns that all relevant isoforms are deleted.

One way to modulate the function of the ARF regulatory network is through the differential splicing of key regulatory domains, outside of the Sec7 domain, such as the lipid-binding Pleckstrin homology (PH) domains of GEFs and GAPs. Phospholipids are critical determinants of membrane identity and orchestrators of membrane trafficking by the recruitment of preferential interactors to specific membrane areas [50]. As such, PH domains can provide substrate specificity by determining membrane localisation or activity in response to certain lipids. The EFA6, BRAG, and Cytohesin (CYTH) families of GEFs, as well as the ADAP, ASAP, ACAP, ARAP, and AGAP families of GAPs contain one or more PH domains [1].

Preferential interaction of certain GEFs and GAPs for distinct lipid species has been described for ARF GEF and GAP splice variants. An illustrative example is the case of the Cytohesin (CYTH1–4) family of GEFs. CYTHs can preferentially bind to either PI(3,4,5)P_3_ or PI(4,5)P_2_, depending on the inclusion or exclusion of a single Glycine microexon within their PH domain. The diglycine (2G) variant of CYTH shows preferential binding to PI(3,4,5)P_3_ and the triglycine (3G) to PI(4,5)P_2_ [51,52]. Functionally, the inclusion or skipping of this single microexon can result in the inverse effect of CYTH2 (ARNO) and CYTH3 (GRP1) on β1-integrin recycling and adhesion [53]. To this end, the PI(3,4,5)P_3_-interacting isoform of CYTH1^2G^, but not the PI(4,5)P_2_-interacting CYTH1^3G^, promotes ARF6-mediated migration of HeLa cells downstream of c-MET activation by spatially restricting CYTH1^2G^ recruitment and ARF6 signalling to the PI(3,4,5)P_3_-enriched leading edge [54]. This suggests that one major mechanism to direct ARF modules to distinct membrane domains, or even perhaps microdomains, is the differential recruitment of alternate isoforms containing such discriminating lipid specificities.

In polarised epithelial cells, different phosphoinositides localise to distinct intracellular and cell surface domains [55–57]. For instance, while PI(4,5)P_2_ is found asymmetrically on the cell surface, PI(3,4,5)P_3_ is polarised to the basolateral domains [55]. This asymmetry is controlled by the PTEN phosphatase, which removes the 3-phosphate group from PI(3,4,5)P_3_ to produce PI(4,5)P_2_ [56]. PTEN is frequently perturbed — mutated, down-regulated, or lost — in cancer [58], suggesting that an imbalance in this pathway is a core part of tumourigenesis. In our recent paper, we described that PTEN-null cells become dependent on a CYTH2-ARF6-AGAP1 module to regulate active β1-integrin recycling to promote invasion [47] (Figure 3b). PI(3,4,5)P_3_ localised to the front of cellular protrusions to drive collective invasion. Notably, it is the PI(3,4,5)P_3_-interacting CYTH2^2G^ isoform, not the PI(4,5)P_2_-interacting CYTH2^3G^ isoform, that stratifies patient survival. In addition, AGAP1 is also alternately spliced in its PH domain, resulting in isoforms with differential affinity for phosphatidylserine (PS), a phospholipid particularly enriched at the cell surface. The AGAP1-Long splice variant, which has decreased affinity for PS, could robustly drive invasive activities, while the AGAP1-Short variant (increased PS affinity), possessed more modest invasion-driving activity. Collectively, this demonstrates how alternate splicing of the lipid-interacting regions of ARF GEFs and GAPs can induce drastically different ARF module function outcome, by determining the microdomains at which these ARF modules can act. Importantly, ARFs can bind and stimulate the activity of both PI4-kinases and PI5-kinases [5,12], kinases that act sequentially in the production of PI(4,5)P_2_. This suggesting that ARFs are intimately linked to lipid metabolism, both producing and responding to distinct patterns of phosphoinositide production.

## ARF GEFs and GAPs as signal-integrating hubs

**Figure 4.**
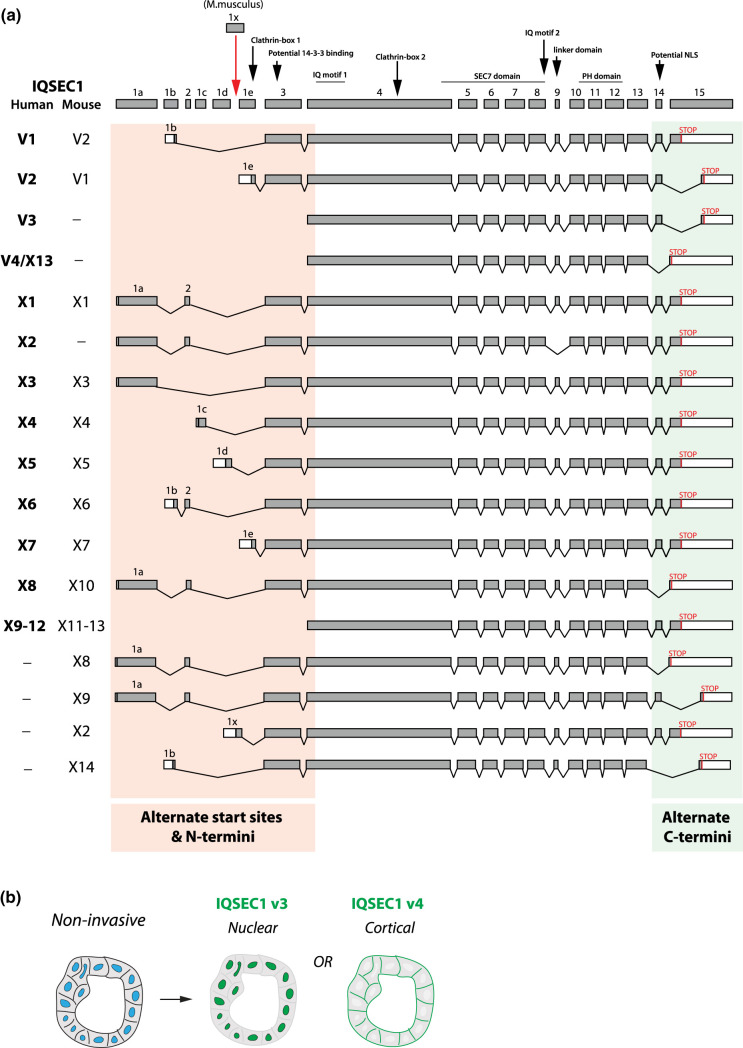
An example of extensive alternate isoforms of an ARF GEF: IQSEC1. (**a**) Comparison of the genomic and domain structure of the ARF GEF IQSEC1 between human and mouse. Alternate start sites result in a range of alternate N-termini (pink). Alternate splicing of the C-terminus results in three alternate C-termini (green). Note lack of alteration in Sec7 or PH domains. (**b**) Different localisations of the ARF GEF IQSEC1 and the phenotypic outcome. Note that neither nuclear nor strict plasma membrane localisation of IQSEC1 conferred by different IQSEC1 variants (in green) drives invasion.

In addition to their conserved catalytic and PH domains, all ARF GEFs and GAPs have additional multifunctional domains. This suggests that more than simply being regulators of ARF nucleotide binding, these act as signal-integrating hubs, precisely controlling where and in response to which signal ARF activity should occur. In addition to changing lipid specificities through the PH domain splicing described above, alternate isoforms changing these additional domains outside the Sec7, ARFGAP, and PH domains can strongly influence the function of these ARF regulators. One demonstration of this is the large number of alternate transcripts of the IQSEC1/BRAG2 GEF (Figure 4a).

In prostate cancer cells, we identified IQSEC1 was required downstream of the ligand HGF and receptor c-Met for *in vitro* invasive, and *in vivo* metastatic, activity [59]. IQSEC1 can be a GEF for both ARF5 and ARF6 [49], and our data suggest that IQSEC1 may regulate these ARFs at distinct spatial locations: ARF6 may act at the tip of invasive protrusions while ARF5 acts in the endosomes just below this invasion tip. Multiple transcripts of IQSEC1 occur through combinations of alternate start codons and exon splicing resulting in combinations of distinct N- and C-termini (Figure 4a). Notably, c-Met associated with all variants, none of which are altered in their Sec7 or PH domains, allowing all variants in theory to be capable of exchange activity on ARFs. Despite this, alternate variants of IQSEC1 possessed highly different localisations and functional effects on collective cell behaviour (Figure 4b). The shortest IQSEC1 variant (v4) lacking N-terminal extension but having a short C-terminal extension after the PH domain localised largely to the cell surface. A distinct C-terminal sequence IQSEC1 variant 3 (v3) confers a nuclear localisation sequence (NLS) which was dominant over the PH domain, as the majority of this variant localised to nuclei (IQSEC1 nuclear localisation and function has been reported). Notably, neither of these variants were capable of robust stimulation of invasion, indicating that invasion is not simply related to whether the GEF can localise to the cell surface or not, or their ability to interact with c-Met. Rather, a specific variant (variant 2) possessed an N-terminal extension, which resulted in it localising to the tips of invasive protrusions. Mechanistically, we mapped this unique function of IQSEC1 v2 to the ability to bridge c-Met to the endocytic co-receptor LRP1, which was required for the efficient internalisation of c-Met and appropriate downstream signalling. A key effector of ARF5/6 in this process is the PI(4,5)P_2_-producing PIP5K1B, providing the substrate for PI(3,4,5)P_3_ production. In turn, this restriction of IQSEC1-ARF activity to invasive protrusions results in PI(3,4,5)P_3_ enrichment at the tips, driving invasion (Figure 3b). In this example, alternate isoforms of the same GEF have opposing functions in the cells, emphasising that consideration of which isoforms are present is essential to elucidating how a GEF or GAP functions. Therefore, alternate GEF and GAP isoforms can utilise the same GTPase in different ways, by controlling the subcellular site at which membrane domains the regulator can bind, or by coupling the regulator to alternate binding partners.

## Conclusion

In recent years, the ARF GTPases are re-emerging as regulators of key cellular behaviours, including lipid metabolism, tumour formation, metastasis, and cross-talk with the immune system. This review depicts but a few of the multiple layers of regulation that can occur in ARF GTPase signalling. We emphasise that phenotypic screening, proteomic profiling of non-mutant GTPases, consideration of splice variants, and *in vivo* experimentation are important tools in the experimental arsenal, in addition to fundamental biochemical *in vitro* approaches. Collectively, a seeming resurgence in interest in ARF GTPases in recent years, such as the following non-exhaustive example list [13,21,33,42,43,47,59], may help to elucidate their function and potential targetability in human disease.

## Perspectives

- The ARF GTPase family is also comprised of the ARF-Like (ARL), the secretion-associated Ras-related (SAR), and tripartite motif-containing protein 23 (TRIM23) GTPases [1]. The function of many of these additional family member GTPases is poorly understood. The extent to which ARL, SAR, and TRIM23 proteins and their regulators are also regulated by the considerations above is an open question. Mutations in some members of the ARL subfamily (e.g. ARL3, ARL6, ARL13B) are associated with ciliopathy diseases [1], emphasising that there is still a wealth of clinically relevant cellular mechanisms to undercover in the ARF family.
- In addition to the mechanisms described above, the ARF regulatory network is likely to be regulated by extensive post-translational modifications. For instance, in addition to splicing in the PH domain determining lipid specificity for CYTH GEFs [51–54], phosphorylation of the PH domain provides an electrostatic switch to rapidly control dissociation from membranes [60]. Such modifications also constitute largely unexplored mechanisms likely to expand the signals that control ARF regulation.
- ARF GTPases are emerging as key regulators in lipid and energy homeostasis [33,61,62], which appears to be fundamental in the cross-talk between tumour and stromal immune cells [13], raising the possibility that this might be exploited as a cancer therapeutic. Most studies on ARFs have focused on cell autonomous trafficking pathways, however, *in vivo* studies on ARF1 in Drosophila melanogaster indicate that this Class I ARF regulates cross-talk between the gut epithelium and intestinal microbiota [61,63]. Understanding how cells regulate communication between different cell types is still in its infancy but may be key to identifying and potentially targeting *in vivo* ARF functions.

## Open Access

Open access for this article was enabled by the participation of University of Glasgow in an all-inclusive *Read & Publish* agreement with Portland Press and the Biochemical Society under a transformative agreement with JISC.

## Abbreviations

3D: 3-dimensional
AGAP: ArfGAP with GTPase domain
ARF: adenosine diphosphate regulatory factor
CYTH: Cytohesin
ECM: extracellular matrix
GAP: GTPase-activating protein
GEF: guanine nucleotide exchange factor
GTPase: guanine triphosphatase
HGSOC: high-grade serous ovarian cancer
PH: Pleckstrin homology
PI(3,4,5)P_3_: phosphatidylinositol-3,4,5-*tris*-phosphate
PI(4,5)P_2_: phosphatidylinositol-4,5-*bis*-phosphate
PTEN: phosphatase and tensin homolog
WT: wild-type
